# Solve the post-operative subdural pneumatosis of chronic subdural hematoma: A novel active bone hole drainage system

**DOI:** 10.3389/fneur.2022.969955

**Published:** 2022-09-01

**Authors:** Sheng Zhang, Xin Zhang, Jian Ding

**Affiliations:** ^1^Department of Neurosurgery, Qingpu Branch of Zhongshan Hospital Affiliated to Fudan University, Shanghai, China; ^2^Nanjing Medical University, Nanjing, China

**Keywords:** chronic subdural hematoma, drilling and drainage, active bone hole drainage system, post-operative subdural pneumatosis, modified surgery

## Abstract

**Background:**

Post-operative subdural pneumatosis (PSP) is commonly encountered after the chronic subdural hematoma (CSDH) surgery which currently lacks effective methods to avoid the condition. This study invented an active bone hole drainage system to change the venting technique with the aim of comparing post-operative efficacy and prognosis to traditional drilling and drainage.

**Methods:**

We conducted a randomized controlled trial between January 2020 and January 2021. A total of 86 patients undergoing surgery were assessed for eligibility, with 50 patients randomly assigned to the control group (received drilling and drainage) and 36 patients to the test group (received modified surgery). The 6-month follow-up was done after surgery. CSDH recurrence and post-operative hematoma re-increasement were the primary endpoints. The data from the two groups were compared and analyzed. This study was registered with the Chinese Clinical Trials Register (ChiCTR2200057158), and had ethics committee approval and patient consent.

**Results:**

The incidence of PSP in the test group (0%, 0/30)was lower than the control group (93.88%, 46/49) (*P* < 0.001). The brain non-expansion rates 3 days/weeks/months after surgery of the test group were 59.25 [49.62, 76.97], 52.10 [42.88, 72.45], and 29.45 [23.40, 36.95] respectively, which were lower than the control group which were 78.60 [69.50, 94.70], 73.10 [60.70, 87.40], and 61.70 [51.50, 78.30], respectively (*P* < 0.001). The ADL scores a week/month/3/6 months after surgery of the test group were 100.00 [60.00, 100.00], 100.00 [85.00, 100.00], 100.00 [100.00, 100.00], 100.00 [100.00, 100.00], which were better than the control group's 60.00 [60.00, 80.00], 75.00 [60.00, 100.00], 100.00 [60.00, 100.00], 100.00 [60.00, 100.00] (*P* < 0.05). The incidence of primary endpoints in the test group (10%, 3/30) was lower than the control group (34.69%, 17/49) (*P* < 0.05).

**Conclusions:**

Compared to drilling and drainage, the modified surgery with the active bone hole drainage system significantly reduced the incidence of PSP and primary endpoints and improved the post-operative efficacy and prognosis.

**Clinical trial registration:**

Identifier: ChiCTR2200057158.

## Introduction

**Figure 1:**
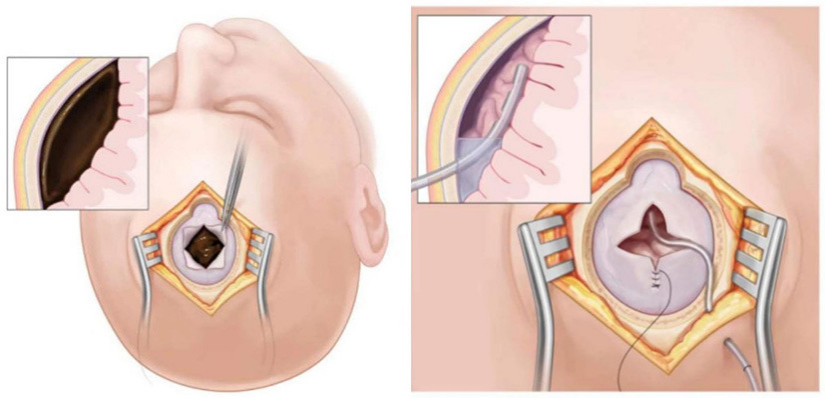
Schematic diagram of drilling and drainage.

Chronic subdural hematoma (CSDH) is a prevalent neurosurgical condition that commonly affects the elderly. In many cases, the neurological function may be significantly reduced, resulting in the loss of ability to take care of themselves (1–3). Surgery is required in most cases of CSDH. Drilling and drainage are among the most extensively used and accepted surgeries (4, 5). Figure 1 indicates the operation in a non-sealed state for flushing and drainage (6). The brain tissue re-expansion effect of most elderly groups is poor, and there are few routines and effective venting processes during the operation. Hence, post-operative subdural pneumatosis (PSP) is a common condition that occurs after the surgery (7, 8). PSP is linked to a poor prognosis, such as recurrence of CSDH (9–11). However, the current dilemma is that, except for the surgical experience of senior physicians, there are no suitable auxiliary means to effectively avoid the above risk more feasibly.

This study improved the traditional drilling and drainage with the innovatively invented active bone hole drainage system to overcome the issue of massive PSP. This technology has not been explored much and no similar technology has been reported in the literature. This study aims to provide novel concepts and methods for the diagnosis and treatment of CSDH.

## Methods

### Patients

**Figure 2:**
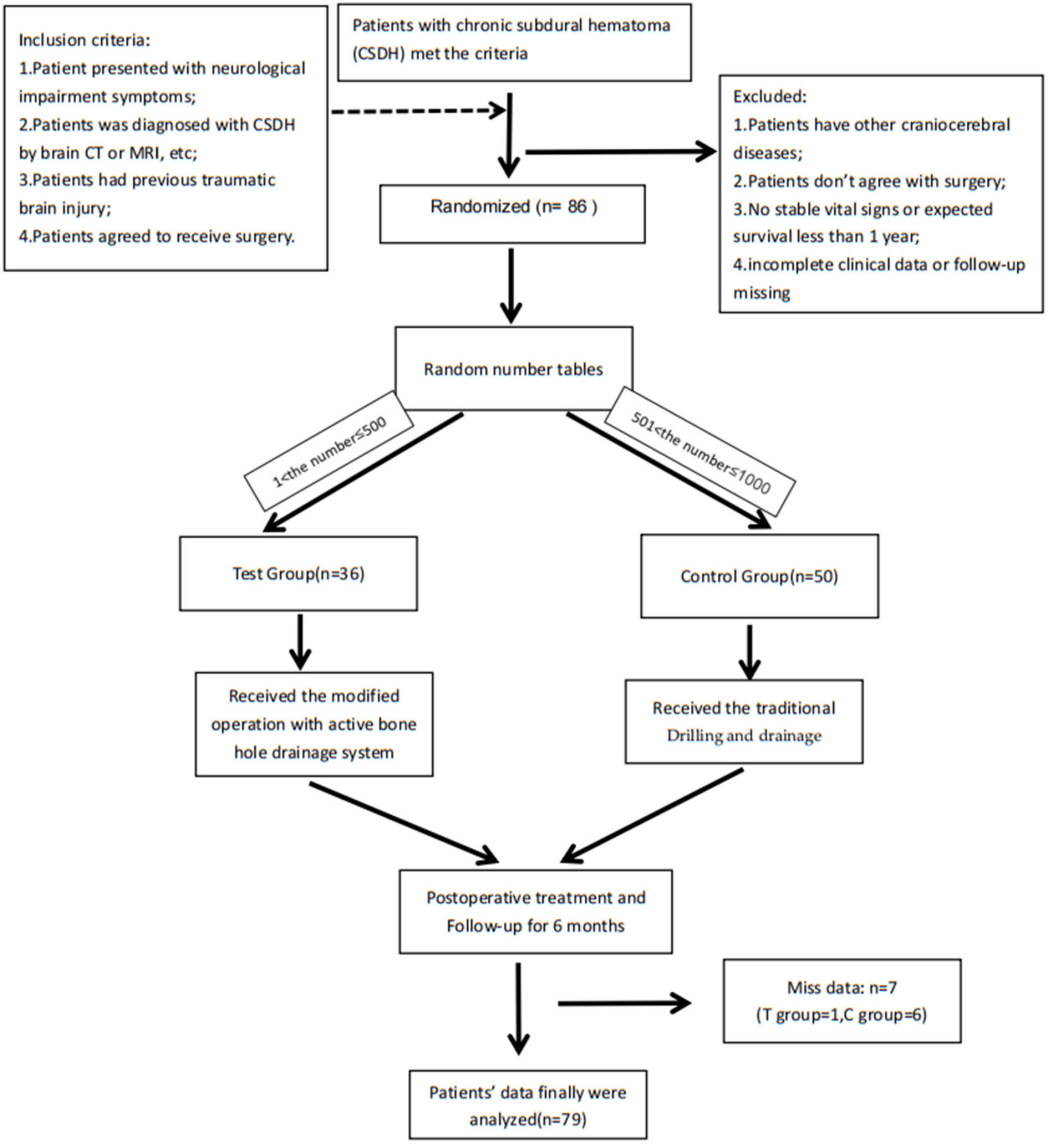
Flow chart of the whole research: Patients were randomly assigned by random number tables: the C language-rand () function randomly generates a number between 1 and 1,000, they were assigned to the control group (received the traditional surgery) when 1 < number ≤ 500 and they were assigned to the test group (received the modified surgery) when 501 < number ≤ 1,000. After the operation, residents took over post-operative care. The residents and patients did not know the group assignment and surgery conditions other than surgeons, while surgeons didn't participate in the treatment and didn't know the patients' hospitalization information. Only the project manager had all the information. This study was registered with the Chinese Clinical Trials Register (ChiCTR2200057158).

Patients admitted to the Neurosurgery Department of hospital with chronic subdural haematoma for surgery were assessed for eligibility between January 2020 to January 2021. They were randomly assigned by random number tables to the control group (received the traditional surgery) and to the test group (received the modified surgery), shown in Figure 2. The nature of intervention did not allow for masking of treatment allocation. However, data were anonymised and clinicians were masked to outcomes when possible. Inclusion criteria: (1) The patient presented with neurological impairment symptoms such as dizziness, headache, vomiting, memory loss, etc.; (2) The patient was diagnosed with chronic subdural hematoma by brain CT or MRI and other imaging examinations; (3) The patient had previous traumatic brain injury; (4) The patient agreed to receive surgical treatment and signed the informed consent. In order to avoid confounding factors, we excluded the following patients: (1) Patients have other craniocerebral diseases (cerebral hemorrhage, brain tumor, cerebrovascular malformation, etc.); (2) Patients who do not need to accept or do not agree to receive surgery; (3) No stable vital signs or expected survival <1 year; (4) CSDH was divided into some or many separate parts with one or some membranes, because it may need neuroendoscopy or other methods.

### Patient management

After patients were admitted, the relevant pre-operative preparations including doctor-patient communication and informed consents were immediately completed. Surgery was performed under general anesthesia. After surgery, patients were returned to the intensive care unit for 1 day and then transferred to the general ward for family accompaniment. Related complications were prevented and the daily drainage volume was noted. Drainage tubes were removed in both groups at about 72 h after operation. In all groups, a head CT was conducted immediately after surgery and again 72 h later. Head CT scans were performed 1 week, 1, 3, and 6 months after the surgery. CSDH recurrence and post-operative hematoma re-increasement (PHRI) were the primary endpoints. CSDH recurrence was defined as post-operative subdural hematoma re-accumulation with severe neurological deficit symptoms requiring reoperation (12, 13). A post-operative hematoma absorption >50% followed by a rise was described as PHRI. However, it did not cause acute and apparent symptoms that would need an emergency re-operation.

### The traditional drilling and drainage

The patient is placed in the supine position with the head turned to the unaffected side. The scalp projection point corresponding to the thickest part of the hematoma is taken and an about 3–5 cm longitudinal incision is made. Also make sure that the incision is at the highest point of the body position. The patient is given endotracheal intubation and general anesthesia. After incision, a bone hole is drilled, the endocranium is incised crosswise and cauterized with bipolar coagulation. One end of the subdural drainage tube is connected to saline, and the other accesses into the subdural space in all directions to irrigate until the effluent runs clear. A soft silicone tube is routinely inserted into the subdural space and connected to sterile drainage bag (Figure 1). Finally, the incision is closed to end the operation.

### The active bone hole drainage system

**Figure 3:**
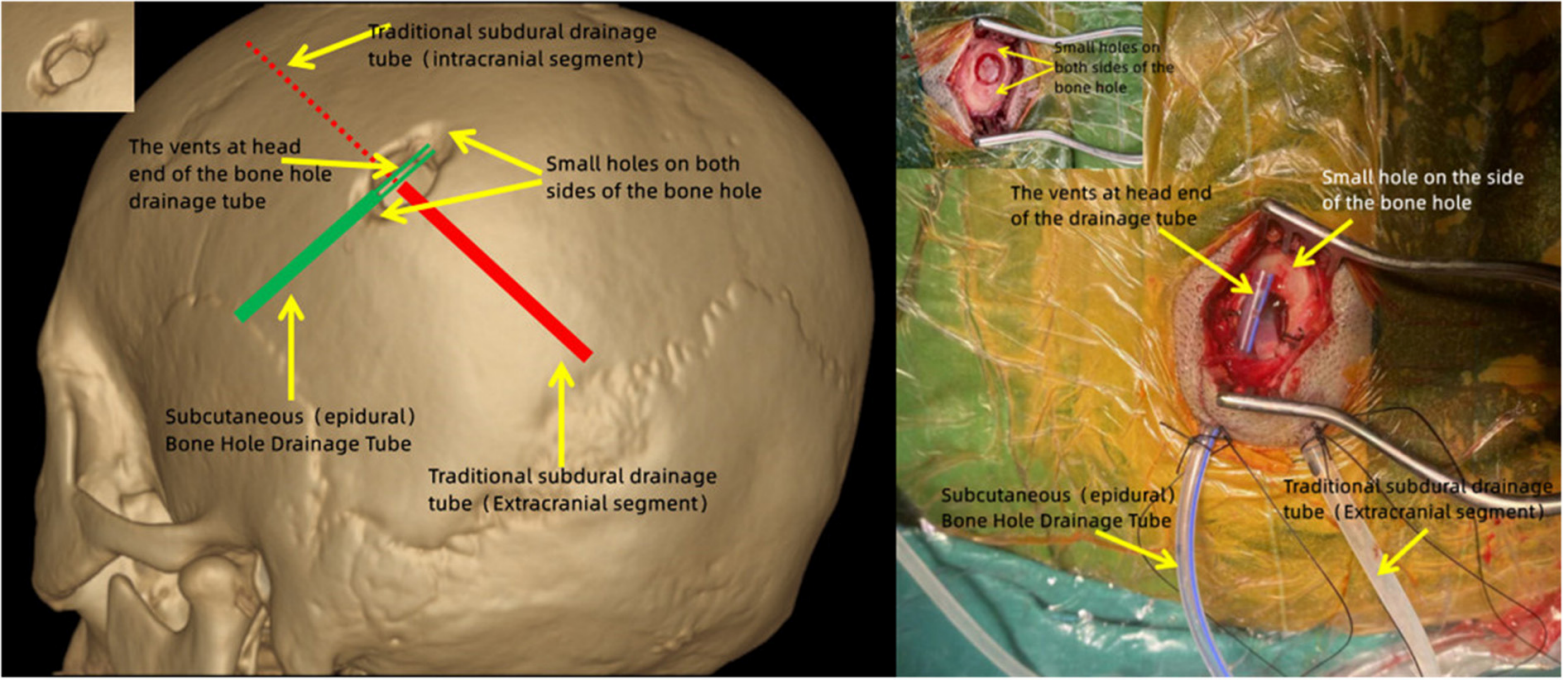
Schematic diagram of the active bone hole drainage system.

The system aims to solve the PSP problem. Based on only one subdural drainage tube after traditional drilling and drainage, the principle was to add a bone hole drainage tube (subcutaneous and epidural) to remove PSP. The specific procedure is as follows: (1) After regular irrigation and the subdural drainage tube is placed, two small bone holes (about 1 × 0.5 cm, respectively, slightly smaller than the original one) are made on both sides of the bone hole (longitudinal direction); (2) Place and immobilize the head end of the bone hole drainage tube into two small bone holes, which directly faced the bone hole (Figure 3). (3) Close the incision to turn an open environment into a airtight one. Then the venting process can be started: (1) Reconfirm the body position, ensuring the bone hole is at the highest point; (2) Continue to irrigate saline into the subdural space through the subdural drainage tube, and the water pressure will push the subdural air out of the subdural space and discharge air through the bone hole drainage tube; (3) After confirming with the anesthesiologist, hold up the head and shake it to physically exhaust the air bubbles attached to the surface of the brain and the inner surface of the skull; (4) Complete the venting procedure when no visible air bubbles are continuously discharged from bone hole drainage tube. To summarize, an effective and adequate venting process is carried out through body position/hydraulic venting/physical venting to reduce the risk of massive PSP despite no obvious increasement in the surgical trauma.

### The follow-up

Following discharge, the patients were instructed to follow up in the neurosurgery outpatient department at 1, 3, and 6 months following the operation, and a head CT examination was performed, respectively. The major goal of follow-up is to see if the primary endpoints (CSDH recurrence and PHRI) occur, as well as if the recovery of patients is comprehensively assessed by using the Activity of Daily Living Scale (ADL) (14).

### Clinical variables obtained

**Figure 4:**
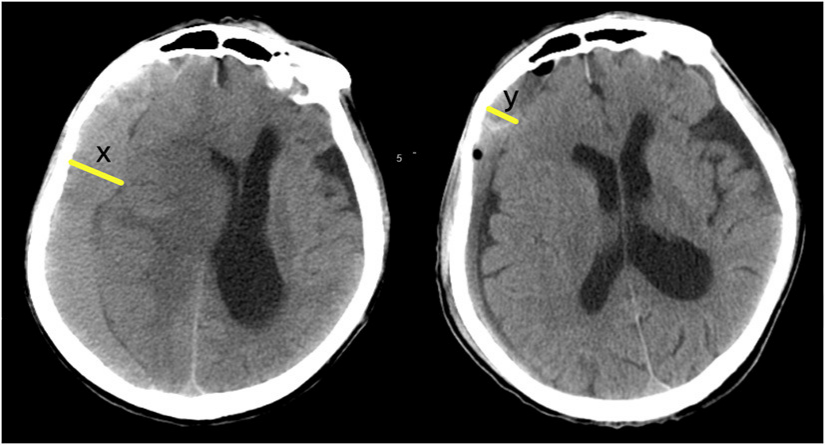
Post-operative BNER: x = maximum width of pre-operative hematoma; y = maximum width of residual hematoma at a certain time point after operation (3 days or a week, or a month after surgery). BNER = y÷x. The greater the value, the worse the degree of brain redilatation.

(1) Basic patient information included gender, age, history of anticoagulant or antiplatelet drugs, history of hypertension, diabetes, or tobacco and alcohol consumption, pre-operative ADL scores, brain atrophy indexes [Evans index (EI) (15), and Cortical atrophy scale (CAS) (15)], hematoma-related indexes [hematoma side, surgical side, hematoma volume, maximum hematoma thickness, midline shift, hematoma density (16), and classifications including the homogeneous/the laminar/the separated/the trabecular type (17)], and biochemical blood indexes (pre-operative albumin and serum sodium, and mean albumin and mean serum sodium, 1 week after operation).

(2) PSP, CSDH recurrence, and PHRI were among the complications indices, where PSP is defined in this study as the volume of air in the operative area (subdural space) >3 ml on post-operative CT scans. Volumetric analysis was performed using the Mimics l7.0 (Materialize Co., Ltd, Beijing, China). The hematoma area or air was manually delineated in multiple image slices for each CT scan, and then the software interpolation was utilized to provide a volumetric representation of the hematoma or air.

(3) Prognostic indexes included the brain non-expansion rates (BNER) 3 days/week/month after surgery and the ADL scores a week/month/3/6 months after surgery (Figure 4).

### Statistical analysis

SPSS Statistics 26 (IBM Corp.), R3.5.0 (rproject.org) was used for all statistical analyses. The continuous variables that did not have a normal distribution were represented by quartiles, and non-parametric tests were performed. The significance level was set at α = 0.05. Categorical variables were analyzed using Pearson's chi-square test or Fisher's exact test and expressed as a percentage of count variables. Cox regression analysis was used to compare the effect of the control group and the test group in predicting primary endpoints.

### Outcomes

#### Comparison of baseline characteristics between test group and control group

A total of 86 CSDH patients were included based on the inclusion and exclusion criteria. A total of 50 patients were randomly allocated to the control group, and 36 patients were allocated to the test group. Among them, seven patients dropped out during the follow-up due to unscheduled outpatient visits, death, and other reasons. Finally, we obtained the complete clinical data and follow-up data of 79 patients (control group: 49; test group: 30) (Table 1).

**Table 1:** Baseline characteristics between the test group and control group.

**Variables**	**Test group (*n* = 30)**	**Control group (*n* = 49)**	***p*-value**
Age (years) median [IQR]	69.00 [64.25, 78.25]	76.00 [67.00, 82.00]	0.075
**Gender (%)**			0.620
Male	26/30 (86.67)	39/49 (79.59)	
Female	4/30 (13.33)	10/49 (20.41)	
**History of drugs**, ***n*** **(%)**			0.650
No	27/30 (90.00)	41/49 (83.67)	
Yes	3/30 (10.00)	8/49 (16.33)	
**History of hypertension**, ***n*** **(%)**			0.943
No	12/30 (40.00)	20/49 (40.82)	
Yes	18/30 (60.00)	29/49 (59.18)	
**History of diabetes mellitus**, ***n*** **(%)**			0.535
No	25/30 (83.33)	38/49 (77.55)	
Yes	5/30(16.67)	11/49 (22.45)	
**History of tobacco and alcohol, n (%)**			0.125
No	10/30 (33.33)	25/49 (51.02)	
Yes	20/30 (66.67)	24/49 (48.98)	
Evans index	26.05 [24.38, 27.28]	26.00 [25.10, 27.40]	0.521
**CAS**			0.685
1	9/30 (30.00)	9/49 (18.37)	
2	12/30 (40.00)	24/49 (48.98)	
3	6/30 (20.00)	11/49 (22.45)	
4	3/30 (10.00)	5/49 (10.20)	
Hematoma volume (ml) [mean (SD)]	117.17 (32.69)	124.93 (36.22)	0.341
**Hematoma side**			0.270
Left side	15/30 (50.00)	18/49 (36.73)	
Right side	10/30 (33.33)	15/49 (30.61)	
Bilateral hematoma	5/30 (16.67)	16/49 (32.65)	
**Hematoma side**			0.119
Unilateral hematoma	25 (83.33)	33/49 (67.35)	
Bilateral hematoma	5 (16.67)	16/49 (32.65)	
**Surgery side**			0.203
Left side	16/30 (53.33)	24/49 (48.98)	
Right side	13/30 (43.33)	17/49 (34.69)	
Bilateral surgery	1/30 (3.33)	8/49 (16.33)	
**Surgery side**			0.162
Unilateral	29/30 (96.67)	41/49 (83.67)	
Bilateral	1/30 (3.33)	8/49 (16.33)	
Maximal hematoma thickness (cm) [mean (SD)]	2.39 (0.61)	2.50 (0.55)	0.412
**Hematoma density (%)**			0.008
Mixed density	14/30 (46.67)	10/49 (20.41)	
Equidensity	11/30 (36.67)	14/49 (28.57)	
Low density	2/30 (6.67)	19/49 (38.78)	
Slightly high density	3/30 (10.00)	6/49 (12.24)	
**Classifications**			0.715
Homogeneous type	10 (33.33)	17 (34.69)	
Laminar type	5 (16.67)	6 (12.24)	
Separated type	5 (16.67)	13 (26.53)	
Trabecular type	10 (33.33)	13 (26.53)	
Pre-operative ADL scores	80.00 [42.50, 80.00]	50.00 [40.00, 60.00]	0.009
Pre-operative albumin (g/L) [mean (SD)]	40.10 (4.61)	38.95 (5.07)	0.316
Pre-operative serum sodium (mmol/L)	138.63 (3.24)	140.61 (2.94)	0.007
1 week post-operative albumin (g/L) [median (IQR)]	36.00 [34.00, 38.00]	35.00 [33.00, 37.00]	0.152
1 week post-operative albumin (mmol/L)	138.50 [136.25, 140.00]	139.00 [137.00, 140.00]	0.327

P-values of the differences in baseline data between the two groups were more significant than 0.05, indicating no statistically significant difference between them.

#### Comparison of complication indexes and prognostic indexes between test group and control group

The incidence of PSP in the test group (0%, 0/30) was lower than the control group (93.88%, 46/49). The BNERs 3 days/week/month after surgery of the test group were 59.25 [49.62, 76.97], 52.10 [42.88, 72.45], and 29.45 [23.40, 36.95] respectively, which were lower than the control group 78.60 [69.50, 94.70], 73.10 [60.70, 87.40], and 61.70[51.50, 78.30] respectively. The ADL scores a week/month/3/6 months after the surgery of the test group were 100.00 [60.00, 100.00], 100.00 [85.00, 100.00], 100.00 [100.00, 100.00], and 100.00 [100.00, 100.00], respectively, which were higher than the control group's 60.00 [60.00, 80.00], 75.00 [60.00, 100.00], 100.00 [60.00, 100.00], and 100.00 [60.00, 100.00] (Table 2).

**Table 2:** Complication and prognostic indexes between test group and control group.

**Variables**	**Test group (*n* = 30)**	**Control group (*n* = 49)**	***p-*value**
**PSP (%)**			<0.001
Yes	0/30 (0.00)	46/49 (93.88)	
No	30/30 (100.00)	3/49 (6.12)	
BNER 3 days after surgery	59.25 [49.62, 76.97]	78.60 [69.50, 94.70]	<0.001
BNER a week after surgery	52.10 [42.88, 72.45]	73.10 [60.70, 87.40]	<0.001
BNER a month after surgery	29.45 [23.40, 36.95]	61.70 [51.50, 78.30]	<0.001
ADL scores a week after surgery	100.00 [60.00, 100.00]	60.00 [60.00, 80.00]	0.007
ADL scores a month after surgery	100.00 [85.00, 100.00]	75.00 [60.00, 100.00]	0.001
ADL scores 3 months after surgery	100.00 [100.00, 100.00]	100.00 [60.00, 100.00]	0.039
ADL scores 6 months after surgery	100.00 [100.00, 100.00]	100.00 [60.00, 100.00]	0.039

In addition, the P-values of each were all <0.05, indicating a significant statistical difference between the two groups, which means the test group had fewer complications than the control group and its prognosis condition was better than that of the control group.

#### Comparison of the primary endpoints between test group and control group

**Figure 5:**
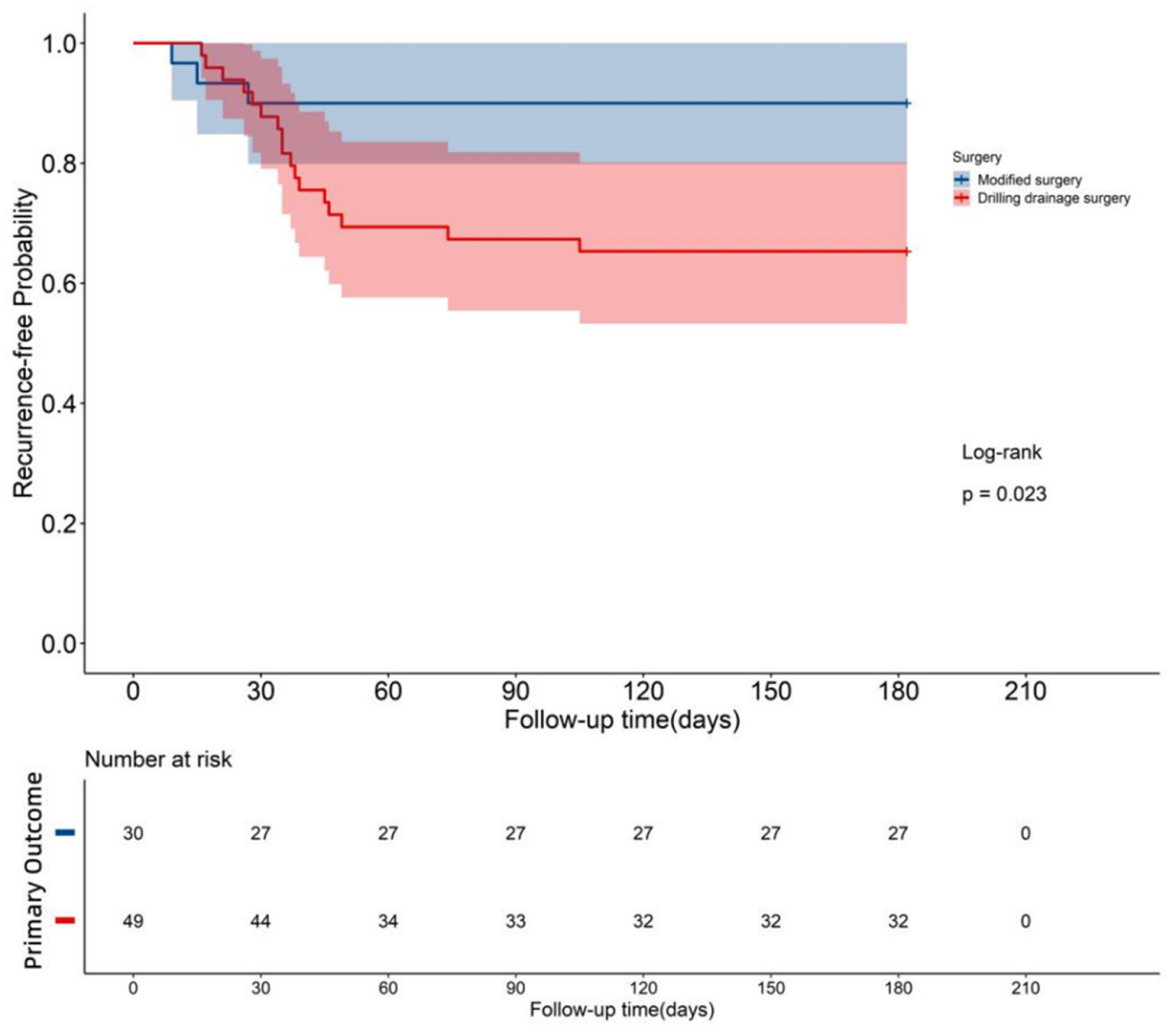
Survival curve: Both groups were monitored for 6 months after the operation, and the survival curves of the two groups were drawn. *P* = 0.023 < 0.05, indicating significant differences in the post-operative survival condition between the two groups. The survival rate of the test group that underwent modified surgery was greater than that of the control group that underwent traditional surgery.

During the 6-month follow-up, the test group had one CSDH recurrence and two PHRI, for a total of three cases of primary endpoints. In the control group, five CSDH recurrence and twelve PHRI were found, and 17 cases of primary endpoints were recorded. There was no significant difference between the recurrence rate of CSDH (3.33%, 1/30 vs. 10.20%, 5/49) and the incidence of PHRI (6.67%, 2/30 vs. 24.49%, 12/49) in the test group and the control group (P > 0.05). However, there was a significant difference between the test group and control group in the total incidence of primary endpoints (10.0%, 3/30 vs. 34.69%, 17/49) (P <0.05). Table 3 and Figure 5 show that the test group had a lower incidence of the primary endpoints in the test group than the control group.

**Table 3:** Primary endpoints between test group and control group.

**Variables**		**Test group**	**Control group**	***P*-value**
*n*		30	49	
CSDH recurrence (%)	No	29/30 (96.67)	44/49 (89.80)	0.496
	Yes	1/30 (3.33)	5/49 (10.20)	
Post-operative hematoma re-increasement (%)	No	28/30 (93.33)	37/49 (75.51)	0.087
	Yes	2/30 (6.67)	12/49 (24.49)	
The total incidence of primary endpoints (%)	No	27/30 (90.00)	32/49 (65.31)	0.029
	Yes	3/30 (10.00)	17/49 (34.69)	

## Discussion

### PSP has a significant adverse effect on prognosis, which has not attracted enough cognition and attention at present

PSP is a common surgical complication of CSDH. There are three primary reasons for its occurrence: (1) The operation necessitates the opening of the subdural space, and the process is not airtight therefore, the air will enter the subdural space; (2) The formation period of CSDH is generally longer than 3 weeks, and the patient's ability to re-expand instantly is restricted after long-term compression of the brain; (3) The main population of CSDH is mostly the elderly, whose proportion of brain atrophy is commonly high and the compliance of brain tissue is usually poor, therefore the brain re-expansion effect is commonly not so good. Henceforth, it is not only prone to subdural air accumulation but also has a large volume. In this study, the incidence of PSP was high in the control group without particular venting intervention, at 93.88%. And the PSP incidence rate (volume ≥ 10 ml) reached 80.4%, and the other (volume ≥ 30 ml) reached 32.61% (as shown in Table 4), which is similar to what has been described in the literature (11).

**Table 4:** The PSP volume of the control group.

**Pneumocephalus volume (PV)**	***N***
PV <10 ml	9
10 ≤ PV <30 ml	22
PV ≥ 30 ml	15
Total	46

Several studies have shown (18–20) that a large amount of PSP seriously affects post-operative efficacy and prognosis of CSDH. The primary mechanism is that a large amount of air accumulates in the subdural space, forming a mass effect that continues to oppress the brain instead of CSDH, preventing brain re-expansion and lengthening the time it takes to re-expand. The control group, which had more post-operative pneumocephalus, had a worse brain re-expansion effect at all time points than the test group that had no apparent post-operative pneumocephalus after the intervention. The prognosis (ADL scores) of all time points was inferior to that of the test group, and the total primary endpoints rate of the control group (34.69%) was higher than that of the test group (10%). So this is the first key point that this study focuses on: PSP is not just a simple risk factor, it has a significant adverse effect on the post-operative efficacy and prognosis of CSDH and is beyond the scope that can be ignored or accepted in clinical practice.

However, the current clinical cognition and attention to PSP are likely insufficient. Firstly, some clinicians believe that a large amount of PSP can be absorbed by ingested without affecting the prognosis. Second, there are only a few related studies that seek to solve the massive PSP problem. Finally, there are no necessary sterile environments and few related possibilities to handle PSP after surgery. Therefore, the optimal moment to tackle the problem is throughout the operation procedure.

### Novel views on the adverse prognosis of CSDH

In previous studies, the primary endpoints were mainly aimed at CSDH recurrence. After reviewing a significant number of CT, MRI, and other imaging data from earlier CSDH cases, we discovered another high-incidence form of post-operative complication that had not been paid enough attention—PHRI. Since there is little literature to distinguish it from CSDH recurrence, this study defines it as After the absorption of residual hematoma was >50%, the hematoma increased again. It and CSDH recurrence are likely to be different stages or outcomes of the same kind of poor prognosis. The most significant difference is that the PHRI does not cause severe acute neurological impairment and does not require emergency surgical intervention (mostly self-absorption), while CSDH recurrence causes significant acute symptoms and requires urgent surgical intervention.

In this study, the respective incidence rates of PHRI in the test group and control group (6.67, 24.49%) were greater than their individual recurrence rates (3.33, 10.2%), and the total PHRI incidence was 17.72% (14/79), not lower than the reported CSDH recurrence rate of 2.5–33% (13, 21–23). And the majority of PHRI patients still have some mild neurological impairment (24). The 6-month follow-up statistics of this study are shown in Table 5. The asymptomatic rate was only 35.7% (5/14).

**Table 5:** The distribution of symptoms in patients with post-operative hematoma re-increasement in this study.

**Symptoms number**	***n***
Mild headache and dizziness	4
Mild nausea and vomiting	2
Mild muscle dysfunction	2
Mild paresthesia	1
Epilepsy	0
No symptoms	5
Total	14

According to the above data, PHR is not “mostly asymptomatic.” Some individuals experience long-term accompanying symptoms or even mild discomfort that can interfere with daily life quality. If there is a way to avoid those poor prognoses, it should be avoided as much as possible, even if it is minor. The venting mechanism in this study is very relevant since it reveals that a considerable amount of PSP can lead to a significant rise in the incidence of PHRI.

Therefore, this is the second primary focus of this study: PHRI is also an important adverse prognosis of CSDH, which needs to be taken seriously and actively dealt with. Despising it or ignoring it is a cover-up, denial of clinically real adverse events, and a lack of professional ethics and medical level.

### Active bone hole drainage system perfectly solved the problem of massive PSP

The goals of surgery development have always been a better minimal invasion, fewer complications, better curative effect, and prognosis. The active bone hole drainage system fully complies with these concepts. Furthermore, few venting methods are in clinical practice (mainly position and subdural flushing) leaving the patient in an open environment where a lot of air can get in (24). However, the system achieves venting in a fully airtight state, proving to be remarkably effective (PSP incidence of test group: 0%). Also, by following the operational protocols of this device, even residents with little clinical experience can achieve a good venting effect. Therefore, the system favors young physicians.

The study's third major finding is as follows: The system perfectly solves the problem of PSP and reduces the incidence of complications and end-point events, resulting in improved efficacy and prognosis.

### Advantages and limitations

The study has the following merits: (1) It addresses the problem of PSP in a scientific and effective way; (2) It is a randomized controlled study with relatively rigorous research methods; (3) The factors included in this study were relatively comprehensive, with a total of 30 indexes; in terms of key treatment indexes, a total of three complications, seven prognosis, and two primary endpoints were thoroughly evaluated; (4) The effect of the system is remarkable. Furthermore, obtaining the necessary equipment is simple. It also does not significantly increase the cost and the trauma of patients. Therefore, it is easy to popularize and apply.

In contrast, this study has several limitations which may cause a bias in the results: (1) This is a single-center study with small sample size; (2) The proportion of bilateral hematomas in the control group (16/49, 32.65%) was higher than that in the test group (5/30, 16.67%). Although the baseline statistics showed no significant difference between the groups (*P* > 0.05), previous studies suggested that bilateral hematoma (25, 26) is a risk factor for recurrence; (3) There are three indexes in the baseline data with significant differences between groups (*P* < 0.05); (4) The definition of PSP in this study is relatively strict (volume > 3 ml), which may lead to a higher incidence compared to previous literature.

## Conclusion

Compared with the traditional drilling and drainage, the modified surgery using the active bone hole drainage system significantly reduced the incidence of post-operative subdural pneumatosis and adverse primary endpoints, and significantly improved the post-operative efficacy and prognosis.

## Data availability statement

The raw data supporting the conclusions of this article will be made available by the authors, without undue reservation.

## Ethics statement

This study was approved by the Ethics Committee of Qingpu Branch of Zhongshan Hospital Affiliated to Fudan University (approval no. IEC-C-007-A08-V.03). The patients/participants provided their written informed consent to participate in this study.

## Author contributions

SZ: concept and design and drafting of the manuscript. SZ and XZ: acquisition, analysis, or interpretation of data, critical revision of the manuscript for important intellectual content, supervision, had full access to all of the data in the study, takes responsibility for the integrity of the data, and the accuracy of the data analysis. XZ: statistical analysis. JD: administrative, technical, or material support. All authors have read and agreed to the published version of the manuscript.

## Funding

This research was funded by the Science and Technology Development Fund Project, grant number QKY2019-11 of the Qingpu District, Shanghai, China.

## Conflict of interest

The authors declare that the research was conducted in the absence of any commercial or financial relationships that could be construed as a potential conflict of interest.

## Publisher's note

All claims expressed in this article are solely those of the authors and do not necessarily represent those of their affiliated organizations, or those of the publisher, the editors and the reviewers. Any product that may be evaluated in this article, or claim that may be made by its manufacturer, is not guaranteed or endorsed by the publisher.
